# Bioactivity-Guided Identification of Anti-Adipogenic Isothiocyanates in the Moringa (*Moringa oleifera*) Seed and Investigation of the Structure-Activity Relationship

**DOI:** 10.3390/molecules25112504

**Published:** 2020-05-28

**Authors:** Linhua Huang, Chunmao Yuan, Yu Wang

**Affiliations:** 1 Citrus Research Institute, Southwest University, Xiema, Beibei, Chongqing 400712, China; huanglh@cric.cn; 2 Citrus Research and Education Center, Food Science and Human Nutrition, University of Florida, 700 Experiment Station Road, Lake Alfred, FL 33850, USA; yuanchunmao01@126.com; 3 State Key Laboratory of Functions and Applications of Medicinal Plants, Guizhou Medical University, 3491 Baijin Road, Guiyang 550014, China

**Keywords:** anti-adipogenesis, isothiocyanates, Moringa seed, structure-activity relationship

## Abstract

Due to the side effects of obesity medications, many studies have focused on the natural products used in the daily diet to control weight. Moringa seed pods and leaves are widely used as vegetables or diet supplements due to the high nutrition value. However, no bioactivity-guided anti-adipogenic study was previously conducted. Therefore, a preadipocyte cell line was adopted as the bioactivity assay to identify the anti-adipogenic compounds in the peeled Moringa seed. Two known sulphur-containing compounds (**1** and **2**) were isolated and identified. Compound **2**, 4-(α-l-rhamnosyloxy) benzyl isothiocyanate, showed a great anti-adipogneic effect with an IC_50_ value of 9.2 μg/mL. The isothiocyanate (ITC) group in compound **2** could be responsible for the inhibitory activity. In addition, a series of compounds with the ITC group were used to further investigate the structure-activity relationship, indicating foods containing ITC derivatives have the potential of being used to control weight.

## 1. Introduction

Obesity is characterised by increased mass in the adipose tissue. In addition to the cosmetic concern, the most serious problem is the increased risk of other diseases, such as cardiovascular disease, type 2 diabetes, and cancers, etc. [1]. Treatments of increasing physical activities and reducing calorie intake are always recommended, and pharmacologic treatment is recommended as a more aggressive way for people who fail to respond to lifestyle interventions [2]. Five anti-obesity medications have been approved by the Food and Drug Administration (FDA), but studies have demonstrated the side effects of these medications [3]. Therefore, screening for the anti-obesity natural compounds in edible food is an alternative safe strategy for preventing obesity [4]. 

*Moringa oleifera* Lam. grows wildly in many tropical and subtropical countries, such as India, Philippines, Cambodia, part of the United States, and the Caribbean Islands [5]. Moringa seeds and leaves contain high levels of nutrients such as vitamins, minerals, and dietary fibres, so they have been used as food sources or dietary supplements. In addition, in some areas, Moringa seeds and leaves are consumed to combat malnutrition in particular iron deficiency [6,7,8]. Extracts from the leaves or seeds were reported to show health benefits such as cholesterol-lowering, anti-diabetic, anti-hypertensive, anti-inflammatory, antioxidant, and anti-hyperlipidaemic effects [9,10,11]. Moringa has been cultivated worldwide as a great commercial crop with fast-growing capacity, easy plantation management, and especially, of nutritive or medicinal value [12]. Hence, this study focused on the chemical composition of the Moringa seed with the anti-adipogenic activity. In the present study, the pre-adipocyte 3T3-L1 cell line, which has the fibroblast-like morphology, has been employed as a bioactivity-guided model combined with analytical techniques (i.e., NMR and LC-MS) to isolate and identify anti-adipogenic compounds from the Moringa seed. In addition, structure-activity relationships were explored. The hypothesis of this study is that some compounds in the Moringa seed are able to inhibit lipid accumulation from preventing adipogenesis, and some specific chemical groups could influence the anti-adipogenic activity. 

## 2. Results and Discussion

### 2.1. Bioactivity-Guided Isolation of Potential Anti-Adipogenic Compounds from Moringa Seeds 

**Figure 1:**
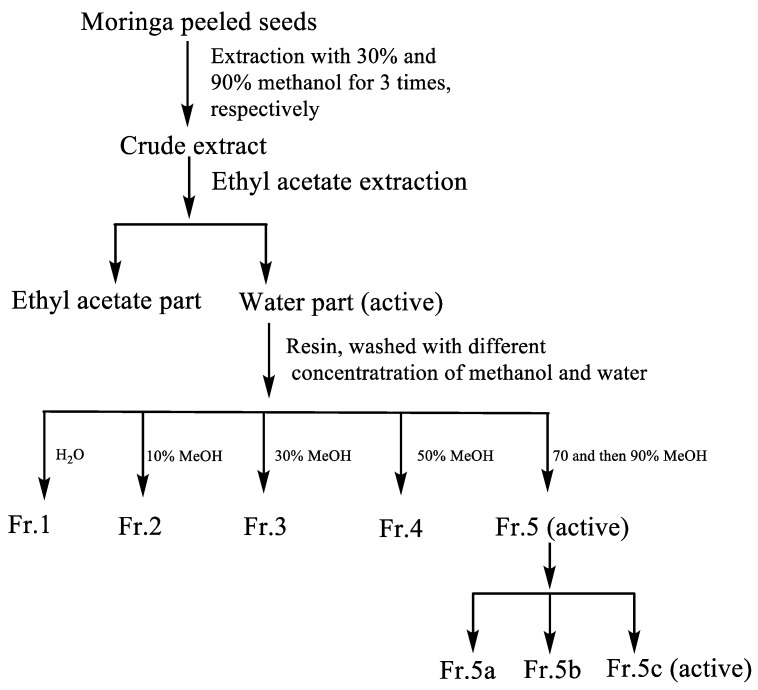
Bioactivity-guided isolation of the anti-adipogenic compounds from Moringa seeds.

**Figure 2:**
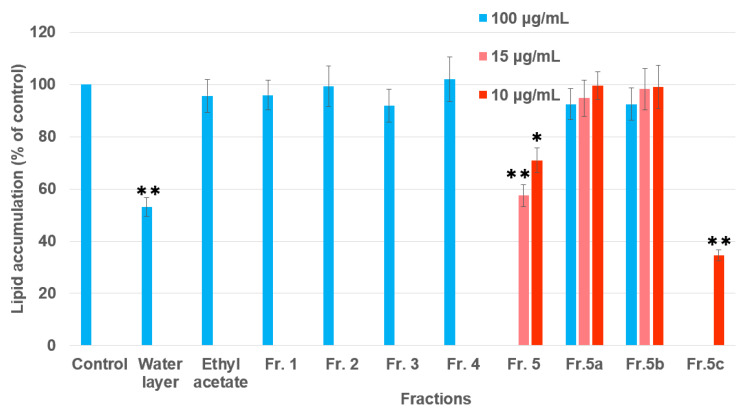
Effect of each fraction isolated from Moringa seeds on lipid accumulation during the differentiation of 3T3-L1 adipocytes. Fr. 5b and Fr. 5c were identified as compounds **1** and **2**, respectively, using LC-MS and NMR. (* *p* < 0.05 vs control, ** *p* < 0.01 vs. control). Data are expressed as mean ± SD (n = 3).

In order to obtain anti-adipogenic compounds from Moringa seeds, each fraction isolated from MeOH/H_2_O extracts was screened for potential anti-adipogenic activity (Figure 1). The active fractions were selected for further isolation. The water layer was subjected to a resin column (SP70) to obtain different fractions, and only fraction 5 (Fr. 5) was active. This fraction was further separated into three parts (Fr.5a–Fr.5c) using a semi-preparative HPLC. The inhibitory effect of each fraction from Moringa seeds on intracellular lipid accumulation is shown in Figure 2 and Table S1. Fr.5c showed the significant inhibitory activity of the intracellular lipid accumulation at 10 μg/mL during 3T3-L1 adipocyte differentiation without a large difference in cell survival rates compared to controls. Fr. 5b and Fr. 5c with single peaks were then identified as compounds **1** and **2**, respectively, using LC-MS and NMR assay. 

### 2.2. Structure Elucidation of Compounds **1** and **2** from Moringa Seeds

**Figure 3:**
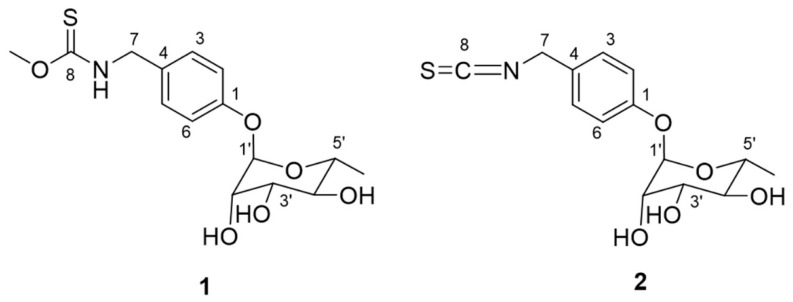
Chemical structures of **1** (niazinin B) and **2** (4-[(α-L-rhamnosyloxy)benzyl]isothiocyanate).

Bioactivity-guided isolation of MeOH/H_2_O extracts of Moringa seeds led to the isolation of two compounds, which were elucidated using spectroscopic methods (1D NMR and LC−Orbitrap−MS). Two compounds, niazinin B (**1**) and 4-(α-l-rhamnosyloxy) benzyl isothiocyanate (**2**) were further confirmed by comparing their spectroscopic data with those reported in the literature [8,13]. The chemical structures of compounds **1** and **2** are shown in Figure 3. All the ^1^H and ^13^C NMR spectra are shown in Figures S1–S4. The LC−MS chromatogram of the isolates (**1** and **2**) in the crude extract is shown in Figure S5. 

### 2.3. Anti-Adipogenic Effects of Purified Compounds 

**Figure 4:**
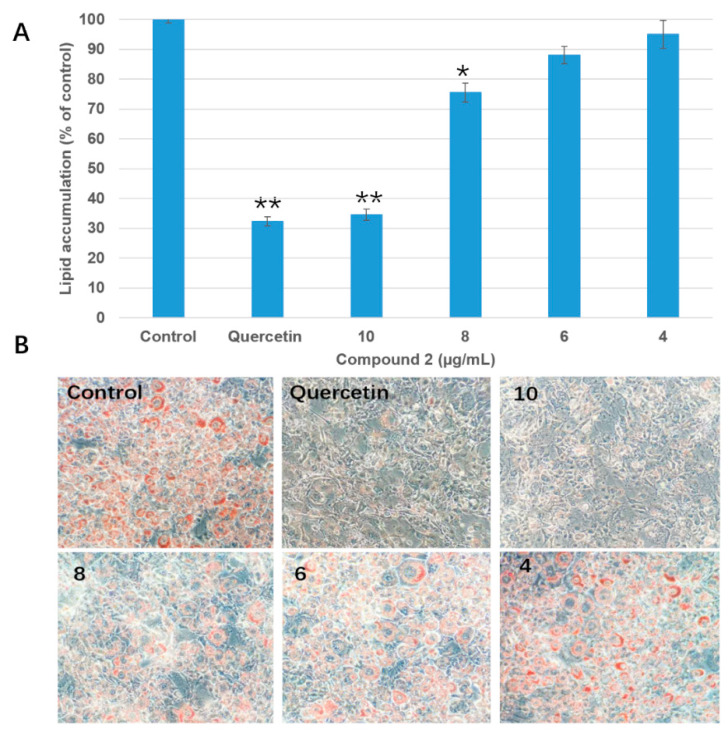
Inhibition of compound **2** on intracellular lipid accumulation. (**A**), Quantification of intracellular lipid using Oil Red O staining assay (* *p* < 0.05 vs. control, ** *p* < 0.01 vs. control). Data are expressed as mean ± SD (n = 3). (**B**), Images of Oil Red O staining 3T3-L1 cells after incubated with different concentrations (10, 8, 6, and 4 µg/mL) of compound **2** taken by an Olympus IX73 microscope (Tokyo, Japan) at 200× magnification. Quercetin (50 µg/mL) was served as a positive control.

**Figure 5:**
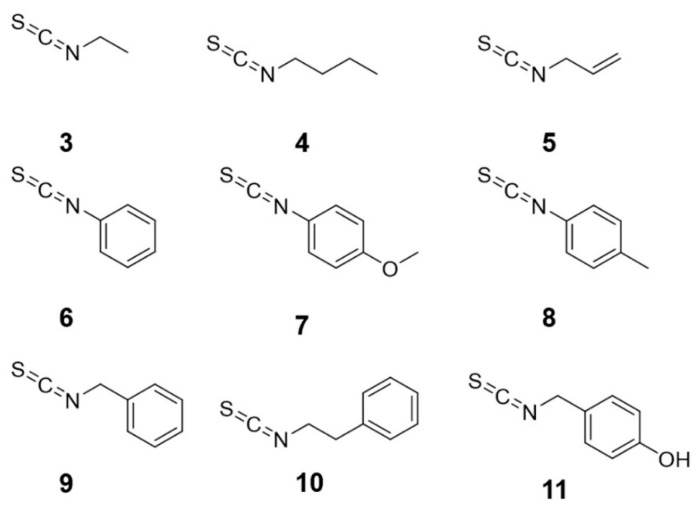
Chemical structures of isothiocyanates (ITCs). **3**, ethyl isothiocyanate, **4**, butyl isothiocyanate, **5**, allyl isothiocyanate, **6**, phenyl isothiocyanate, **7**, 4-methoxyphenyl isothiocyanate, **8**, 4-methylphenyl isothiocyanate, **9**, benzyl isothiocyanate, **10**, phenethyl isothiocyanate, **11**, 4-hydroxybenzyl isothiocyanate.

Both of the isolates (**1** and **2**) were evaluated by measuring the inhibitory effect on lipid accumulation in 3T3-L1 adipocytes (Figure 2). Compound **1** showed no obvious anti-adipogenic activity against 3T3-L1 cells at the concentration of 100 μg/mL. After being differentiated, the intracellular lipid accumulation was significantly inhibited by compound **2** in a dose-dependent manner (Figure 4 and Table S2). The IC_50_ value for the anti-adipogenic ability of compound **2** was 9.2 μg/mL (equal to 29.6 μM) without cytotoxicity, which was better than that of the positive control (quercetin). As shown in Figure 3, both compounds **1** and **2** shared the same skeleton with the benzene ring and glycoside part. The only difference was the presence of an isothiocyanate group in compound **2**, which played a vital role in the anti-adipogenic effect towards 3T3-L1 cells. Benzyl isothiocyanate (BITC, compound **9** in Figure 5), an aryl-ITC obtained from papaya with a similar structure to compound **2**, has been found to restrain body weight gain and liver fat accumulation in high-fat diet-induced mice [14]. BITC has been further demonstrated to inhibit the lipid accumulation significantly (approximately 55% inhibitory ratio) at the concentration of 5 μM incubated with the differentiated 3T3-L1 cells [15]. However, allyl isothiocyanate (AITC, compound **5** in Figure 5), has indicated a much weaker effect on the lipid accumulation compared to that of the BITC reported in the same study [15]. Therefore, in addition to the isothiocyanate group, the anti-adipogenic effect of ITC is closely related to other types of structural group, which has never reported before and are discussed in this study.

### 2.4. Structure-Activity Relationship of Isothiocyanate (ITC) Derivatives

**Figure 6:**
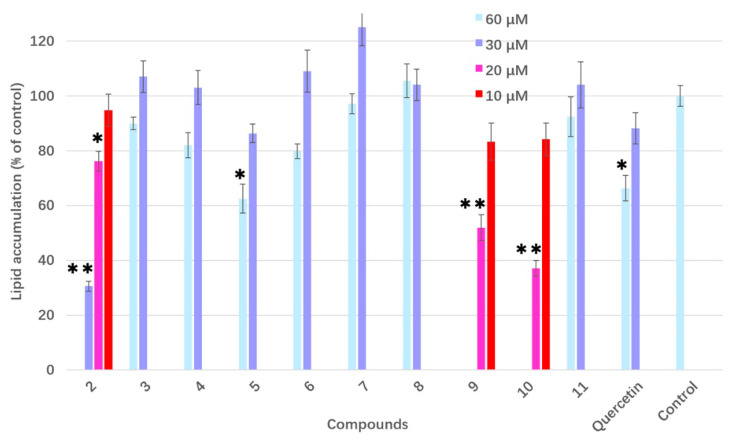
Effects of each ITC on lipid accumulation (* *p* < 0.05 vs. control, ** *p* < 0.01 vs. control). Higher concentrations of compounds **2**, **9**, and **10** showed a significant cytotoxicity. Quercetin was served as a positive control. Data are expressed as mean ± SD (*n* = 3).

In order to illustrate the structure-activity relationship of ITC derivatives, a series of compounds with the ITC group were purchased and employed to make a comparative study on their anti-adipogenic effect using 3T3-L1 cells. The structures of the ITC derivatives were divided into three groups, alkyl or allyl ITC derivatives (compound **3**–**5**), aryl ITC derivatives (**6**–**8**), and arylalkyl ITC derivatives (**9**–**11**), as shown in Figure 5. The anti-adipogenic effect activity of all ITC derivatives, including isolated compound **2** is presented in Figure 6 and Table S3. Quercetin, a commercial anti-obesity flavonoid, was also tested for its anti-adipogenic effect as a positive control. Of the alkyl or allyl ITC group, only compound **5,** with the allyl ITC group, revealed anti-adipogenic activity at a concentration of 60 μM, while other compounds with alkyl ITC group (compounds **3** and **4**) were inactive, which suggested that the double bond attached to the ITC group was important for the anti-adipogenic activity. None of the aryl ITC derivatives (**6**–**8**) was effective at the concentration of 60 μM. Arylalkyl ITC derivatives, **2**, **9**, and **10**, showed significant anti-adipogenic activity. In contrast, compound **11,** with an additional hydroxyl group to the arylalkyl ITC, showed no inhibitory activity of lipid accumulation. Amongst those active arylalkyl ITC derivatives, compound **10** showed the highest effect, indicating longer alkyl chain between the ITC group and benzene ring could enable a positive contribution to their anti-adipogenic activity. Although compound **2** shared the same skeleton as **11**, the activity of those compounds was the opposite. The only difference between those compounds are at the para-position, the benzene ring of compound **2** is connected with rhamnose, while in compound **11** there is a hydroxyl group instead. A hydroxyl group could donate more electron density, which somehow negatively affected the anti-adipogenic activity of compound **11.** In other words, due to the presence of electrophilic aromatic directing groups, the more electron density donated by them, the less anti-adipogenic activity could be observed. Compounds **9** and **10** without the electron donation groups showed better anti-adipogenic activity than that of compound **2**. Therefore, the electron donation effect might decrease the anti-adipogenic activity of arylalkyl ITC derivatives.

In addition, previous studies have indicated that transient receptor potential A1 (TRPA1)-activating potency could be triggered by most of the ITCs, indicating a very slight contribution of other functional groups to the TRPA1-activating ability [16]. However, the length of the side or so-called alkyl chains of ITCs had demonstrated a vital role in their bioactivities. Longer alkyl chain derivatives exert steric effects on antifungal activity of ITCs against *Rhizoctonia solani* but enhancement on their antibacterial activity against *Erwinia carotovora* due to the increased hydrophobicity [17]. As for arylalkyl ITC derivatives, increased alkyl chain length has enhanced the inhibition of an arylalkyl isothiocyanate against nitrosamine 4-(methylnitrosamino)-1-(3-pyridyl)-1-butanone (NNK)lung tumorigenesis [18], which to some extent is in agreement with our conclusion for the anti-adipogenic activity of arylalkyl ITC derivatives. Further work has revealed that high lipophilicity and low reactivity of ITCs might be of importance for the inhibitory activity against NNK-induced lung tumorigenesis. As for the alkyl isothiocyanates, longer alkyl chain could increase the activity for reducing tumor multiplicity. For example, dodecyl isothiocyanates are more active than hexyl isothiocyanates. In this case, the phenyl moiety is not essential for the inhibitory activity against lung tumorigenesis [19]. In addition, the bioactivities of the ITCs derivatives with electron-donating groups have been reported. Compared to “electron-donating group free” phenyl ITC, a decrease in free radical scavenging ability was observed in aryl ITCs bearing the electron-donating groups [20]. However, compared with methyl ITC, an electron donating group to α-carbon (R ± CX1X2 ± NCS) significantly enhanced the glutathione S-transferase (GST) activity, an important enzyme involved in the cellular detoxification of genotoxic and carcinogenic chemicals [21]. 

## 3. Materials and Methods 

### 3.1. Materials

Ethyl isothiocyanate (97%), butyl isothiocyanate (99%), allyl isothiocyanate (99.7%), phenyl isothiocyanate (99%), 4-methoxyphenyl isothiocyanate (98%), 4-methylphenyl isothiocyanate (97%), benzyl isothiocyanate (98%), phenylethyl isothiocyanate (98.5%), and quercetin (>95%) were purchased from Sigma-Aldrich (St. Louis, MO, USA). 4-Hydroxybenzyl isothiocyanate (98%) was purchased from Santacruz Biotechnologies (Santacruz, TX, USA). Thin-layer chromatography (TLC) plates (250 μm thickness, 2−25 μm particle size) were purchased from Macherey–Nagel (Macherey-Nagel, Düren, Germany). Sepabeads resin styrenic adsorbent (130 Å porosity) was obtained from Sorbtech (Peachtree Corners, GA, USA). LC-MS grade acetonitrile, water, and formic acid, and ACS-grade n-hexane, ethyl acetate, acetone, chloroform, and methanol were purchased from Sigma-Aldrich. Moringa seeds collected in West Bengal, India, were purchased from Amazon (Bellevue, WA, USA) in May 2019.

### 3.2. LC-MS Analysis

LC-MS analysis was conducted using a Vanquish ultrahigh-performance liquid chromatography (UHPLC) system combined with a Q Exactive mass spectrometer (Thermo Fisher Scientific, San Jose, CA, USA) via an electrospray ionization (ESI) interface. Chromatographic separations for crude extract and isolates were performed using an ACQUITY UPLC BEH C18 column (150 mm × 2.1 mm i.d., 5 μm, Waters, Milford, MA, USA). All the parameters of LC and MS were referred to tin he methods from our previous work [22].

### 3.3. NMR Analysis

^1^H (400 MHz) and ^13^C (100 MHz) NMR spectra were acquired using a Bruker 400 MHz instrument. Both compounds **1** and **2** were analysed in CD_3_OD. Mest ReNova (Ver. 6.0) was used for the data collection and analysis.

### 3.4. Purification of Compounds **1** and **2**

Peeled Moringa seeds (400 g) were powdered, soaked in MeOH/H_2_O (30%, 2L), and then applied to ultrasonic extraction for three times (2 h for each time) at room temperature. The extracted solution was filtered and concentrated with a rotary evaporator at 40 ℃ to yield crude extracts (74 g). The crude extract was suspended in water and then extracted with ethyl acetate (EtOAc) (3 × 1 L), resulting in the corresponding extracts: EtOAc layer (30 g) and a water layer (32 g). The anti-adipogenic activity was evaluated by measuring the inhibitory ability of adipogenic differentiation in 3T3-L1 preadipocytes. The active water fraction was applied to a macroporous absorption resin (SP70) column chromatography (CC) (7.62 cm × 65 cm i.d.), and eluted with MeOH/H_2_O (0, 30%, 50%, 70%, and 90%, each 3 L) to create five fractions (Fr. 1−Fr. 5). Among the fractions, only Fr. 5 showed better anti-adipogenic activity. Then, Fr. 5 (1.3 g) was further purified by a semi-preparative high-performance liquid chromatography (HPLC) (51% MeOH/H_2_O for 50 min, *v*/*v*, 4 mL/min) with an X-bridge column (250 mm × 20 mm, 5 μm), resulting in three fractions (Fr.5a–Fr.5c). Fr.5b and Fr.5c with retention times of 25.0 and 34.5 min were then identified to be **1** (150 mg) and **2** (230 mg), respectively. Only compound **2** showed good anti-adipogenic activity.

### 3.5. Anti-Adipogenic Activity Assay

The anti-adipogenic activity method was modified from our previous work [22]. Briefly, 3T3-L1 cells (American Type Culture Collection; ATCC CL-193, Rockville, MD, USA) were grown in Dulbecco’s modified Eagle’s medium (DMEM) with the addition of 10% bovine calf serum (BS), 100 units/mL penicillin, and 50 μg/mL streptomycin in an incubator with 5% CO_2_ at 37 °C. 3T3-L1 preadipocytes were differentiated by a mixture of 5 μg/mL insulin, 0.5 mM 3-isobutyl-1-methylxanthine (IBMX), 1 μM dexamethasone (Dex). The medium was replaced with fresh dulbecco’s modified eagle medium (DMEM) supplemented with 10% fetal bovine serum (FBS) and 5 μg/mL insulin twice for every other day. Finally, cells were stained with Oil Red O solution (0.3% Oil Red O in 60% isopropyl alcohol) for 20 min and rinsed twice using PBS. Stained lipid droplets of the 3T3-L1 adipocytes were photographed using an Olympus IX73 microscope (Tokyo, Japan) at 200× magnification. The anti-adipogenic activity was quantified by measuring the isopropyl alcohol dissolved dye retained in the cells at 490 nm using a microplate reader (FlexStation 3, Molecular Devices, Sunnyvale, CA, USA).

### 3.6. Cytotoxicity Assay

The cytotoxic effects of samples on 3T3-L1 cells were measured using a 3-(4,5-dimethylthiazol-2-yl)-2,5-diphenyltetrazolium bromide (MTT) assay modified from Yuan et al. 2019 [22]. All samples tested were dissolved in dimethyl sulfoxide (DMSO) before being applied to preadipocytes. The final concentration of DMSO was no more than 0.2%.

### 3.7. Statistical Analysis

All results of bioactivity measurement were expressed as the mean ± standard deviation (SD) (*n* = 3). Statistical comparisons were determined by Students’ t-test. A *p*-value of <0.05 was considered significant.

## 4. Conclusions

The present study isolated two sulfur-containing compounds from peeled *Moringa oleifera* seeds using a bioactivity-guided assay. The IC_50_ value of the anti-adipogenic ability of compound **2** was 9.2 μg/mL (equal to 29.6 μM) without cytotoxicity, which was better than that of the positive control (quercetin). A series of compounds with the ITC group were purchased and employed to make a comparative study on their anti-adipogenic effect using 3T3-L1 cells. Arylalkyl ITC derivatives (**2**, **9**, and **10**) excepted for compound **11** showed significant anti-adipogenic activity at 20 μM, which might be involved with the electron donation effects in compound **11** decreased the anti-adipogenic activity. The aryl-ITC derivatives (**6**–**8**) did not show any anti-adipogenic activity. To the best of our knowledge, this is the first time that the structure-activity relationship between ITC derivatives and anti-adipogenic activity have been investigated, which provides the potential perspectives of using food or herbs containing compounds with ITC group to control weight and possible chemistry mechanisms.

## Supplementary Materials

The following are available online: Table S1 Effect of each fraction isolated from Moringa Oleifera seeds on lipid accumulation during 3T3-L1 adipocytes differentiation. Table S2 Inhibition of compound 2 on intracellular lipid accumulation during 3T3-L1 cell differentiation. Table S3 Effect of each ITCs on lipid accumulation during 3T3-L1 adipocytes differentiation. Figure S1. The ^1^H NMR spectrum (400 MHz, CD_3_OD) of 1. Figure S2. The ^13^C NMR (100 MHz, CD_3_OD) of 1. Figure S3. The ^1^H NMR spectrum (400 MHz, CD_3_OD) of 2. Figure S4. The ^13^C NMR (100 MHz, CD_3_OD) of 2. Figure S5. Total ion chromatogram (TIC) of the isolates (1 and 2) in *Moringa Oleifera* seeds extract. Figure S6. The HR-ESI-MS spectrum of 1. Figure S7. The HR-ESI-MS spectrum of 2.
