# Supplementary material for: Bioactivity-Guided Identification of Anti-Adipogenic Isothiocyanates in the Moringa (Moringa oleifera) Seed and Investigation of the Structure-Activity Relationship
Source: Molecules. 2020 May 28;25(11):2504. doi: 10.3390/molecules25112504 (PMC7321240; doi:10.3390/molecules25112504)
Supplement: Supplementary file 1 [file molecules-25-02504-s001.pdf]

molecules

Supplementary information

Bioactivity-guided identification of anti-adipogenic isothiocyanates in the Moringa (*Moringa Oleifera)* seed and investigation of the structure-activity relationship

Linhua Huang [1,2,†], Chunmao Yuan [2,3,†] and Yu Wang [2,*]

1 Citrus Research Institute, Southwest University, Xiema, Beibei, Chongqing 400712, P. R. China
2 Citrus Research and Education Center, Food Science and Human Nutrition, University of Florida, 700 Experiment Station Road, Lake Alfred, Florida 33850, United States
3 State Key Laboratory of Functions and Applications of Medicinal Plants, Guizhou Medical University, 3491 Baijin Road, Guiyang 550014, P. R. China
* Correspondence: yu.wang@ufl.edu; Tel.: +1-863-956-8673

† These authors contributed equally to this work..

1D NMR data for compounds **1** and **2**.

Table S1 Effect of each fraction isolated from Moringa Oleifera seeds on lipid accumulation during 3T3-L1 adipocytes differentiation.

Table S2 Inhibition of compound **2** on intracellular lipid accumulation during 3T3-L1 cells differentiation.

Table S3 Effect of each ITCs on lipid accumulation during 3T3-L1 adipocytes differentiation.

Figure S1. The ^{1}H NMR spectrum (400 MHz, CD_3OD) of **1**.

Figgure S2. The ^{13}C NMR (100 MHz, CD_3OD) of **1**.

Figure S3. The ^{1}H NMR spectrum (400 MHz, CD_3OD) of **2**.

Figure S4. The ^{13}C NMR (100 MHz, CD_3OD) of **2**.

Figgure S5. Total ion chromatogram (TIC) of the isolates (**1** and **2**) in *Moringa Oleifera* seeds extract.

Figure S6. The HR-ESI-MS spectrum of **1**.

Figure S7. The HR-ESI-MS spectrum of **2**.

1D NMR data for compounds **1** and **2**.

Niazinin B (**1**) Light yellow oil; HR-ESI-MS (negative ion mode): m/z calcd. for $C_{16}H_{22}NO_8S$ $[M + HCOO]^-$, 388.1066; found, 388.1080. ^1H NMR (400 MHz, CD_3OD):δ 7.04/7.03 (H-2, H-6, d, J = 8.4 Hz), 7.28/7.22 (H-3, H-5, d, J = 8.4 Hz), 4.65 (H_2-7, s), 5.42 (H-1', d, J = 1.8 Hz), 4.00 (H-2', dd, J = 3.5, 1.9 Hz), 3.86 (H-3', dd, J = 9.5, 3.5 Hz), 3.47 (H-4', t, J = 9.0 Hz), 3.64 (H-5', m), 1.23 (H-6', d, J = 6.4 Hz), 3.97 (OMe, s); ^{13}C-NMR (150 MHz,CD_3OD): δ 155.8 (C-1), 116.5 (C-2, C-6),128.8/128.4 (C-3, C-5), 131.7 (C-4), 47.8/45.4 (C-7), 191.8 (C-8), 98.5 (C-1'), 69.2 (C-2'), 70.7 (C-3'), 70.9 (C-4'), 72.5 (C-5'), 16.7 (C-6'), 56 (OMe).

4-(α-L-Rhamnosyloxy) benzyl isothiocyanate (**2**) Light yellow oil; HR-ESI-MS (negative ion mode): m/z calcd. for $C_{15}H_{18}NO_7S$ $[M + HCOO]^-$, 356.0804; found, 356.0818. ^1H NMR (400 MHz, CD_3OD):δ 7.12 (H-2, H-6, d, J = 8.4 Hz), 7.33 (H-3, H-5, d, J = 8.4 Hz), 4.72 (H2-7, s), 5.46 (H-1', br s), 4.02 (H-2', dd, J = 3.3, 1.6 Hz), 3.86 (H-3', dd, J = 9.4, 3.3 Hz), 3.48 (H-4', t, J = 9.6 Hz), 3.64 (H-5', m), 1.24 (H-6', d, J = 6.2 Hz); ^{13}C-NMR (150 MHz,CD_3OD): δ 156.4 (C-1), 116.5 (C-2, C-6),128.3 (C-3, C-5), 128.2 (C-4), 48.0 (C-7), 131.9 (C-8), 98.5 (C-1'), 70.6 (C-2'), 70.8 (C-3'), 72.4 (C-4'), 69.3 (C-5'), 16.6 (C-6').

Table S1 Effect of each fraction isolated from Moringa Oleifera seeds on lipid accumulation during 3T3-L1 adipocytes differentiation.

Sample	Dose (μg/ml)	Lipid accumulation (% of control)[a]	Cell survival rate (% of control)
**Ethyl acetate**	100	95.3 ± 4.3	90.5 ± 4.6
**Water layer**	100	53.6 ± 2.4**	96.4 ± 5.7
**Fr. 1**	100	95.3 ± 4.8	96.7 ± 2.8
**Fr. 2**	100	99.3 ± 5.1	100 ± 2.2
**Fr. 3**	100	91.9 ± 3.8	99.8 ± 1.9
**Fr. 4**	100	102.1 ± 7.4	100 ± 4.2
**Fr. 5**	100		35.7 ± 2.8
**Fr. 5**	15	57.5 ± 3.1**	93.2 ± 2.9
**Fr. 5**	10	71.9 ± 3.6*	91.5 ± 3.8
**Fr. 5a**	100	92.5 ± 4.1	95.7 ± 4.6
**Fr. 5a**	15	94.8 ± 5.4	96.4 ± 3.2
**Fr. 5a**	10	99.5 ± 7.3	100 ± 4.4
**Fr. 5b**	100	93.6 ± 4.9	97.5 ± 5.1
**Fr. 5b**	15	98.2 ± 5.2	101 ± 2.6
**Fr. 5b**	10	99.3 ± 7.0	103 ± 2.9
**Fr. 5c**	100		20.7 ± 3.7
**Fr. 5c**	15		48.6 ± 3.1
**Fr. 5c**	10	37.6 ± 3.7**	90.5 ± 1.9
**Control**	100	100.0 ± 3.2	100 ± 1.6
**Positive Control (Quercetin)**	50	34.5 ± 3.6**	92.4 ± 3.7

Each value is expressed as a mean ± standard deviation (n = 3). **$p < 0.01$ vs. control, *$p < 0.05$ vs. control.

Table S2 Inhibition of compound **2** on intracellular lipid accumulation during 3T3-L1 cells differentiation.

Sample	Dose (μg/ml)	Lipid accumulation (% of control)*	Cell survival rate (% of control)
**2**	10	36.8 ± 3.3**	92.5 ± 4.6
**2**	8	75.5 ± 4.2*	91.4 ± 3.7
**2**	6	88.0 ± 6.5	92.7 ± 3.2
**2**	4	95.2 ± 5.8	99.7 ± 4.5
**Control**	100	100.0 ± 6.7	100.6 ± 3.2
**Positive Control (Quercetin)**	50	35.2 ± 3.2**	94.7 ± 2.8

*Each value is expressed as a mean ± standard deviation ($n = 3$). **$p < 0.01$ vs. control, *$p < 0.05$ vs. control.

Table S3 Effect of each ITCs on lipid accumulation during 3T3-L1 adipocytes differentiation.

Sample	Dose (μM)	Lipid accumulation (% of control) *	Cell survival rate (% of control)
**2**	60		35.6 ± 5.7
**2**	30	30.6 ± 3.1**	92.4 ± 5.3
**2**	20	76.2 ± 4.9*	94.7 ± 3.8
**2**	10	94.8 ± 8.4	95.5 ± 2.8
**3**	60	89.9 ± 6.3	94.8 ± 3.5
**3**	30	107.0 ± 5.8	96.8 ± 4.7
**4**	60	82.4 ± 2.8	98.9 ± 3.8
**4**	30	103 ± 5.7	100 ± 3.9
**5**	60	62.5 ± 4.2*	97.5 ± 3.8
**5**	30	86.3 ± 5.5	98.5 ± 5.3
**6**	60	79.8 ± 4.3	97.4 ± 3.7
**6**	30	109.2 ± 7.1	100.4 ± 5.4
**7**	60	97.1 ± 5.7	97.8 ± 3.6
**7**	30	125.3 ± 6.2	101 ± 4.8
**8**	60	105.5 ± 8.3	100 ± 4.5
**8**	30	104.2 ± 9.1	96.6 ± 4.4
**9**	60		35.8 ± 3.1
**9**	30		47.9 ± 1.9
**9**	20	51.9 ± 3.8**	90.5 ± 2.7
**9**	10	83.3 ± 6.6	97.4 ± 2.4
**10**	60		38.6 ± 4.0
**10**	30		46.6 ± 4.5
**10**	20	37.1 ± 2.5**	92.8 ± 3.4
**10**	10	84.2 ± 5.2	95.5 ± 2.3
**11**	60	92.4 ± 6.7	96.7 ± 4.5
**11**	30	104.2 ± 8.3	100.9 ± 3.6
**Control**	100	100.0 ± 5.1	100 ± 3.2
**Positive Control (Quercetin)**	60	66.3 ± 3.9*	95.5 ± 2.4
**Positive Control (Quercetin)**	30	88.2 ± 6.1	96.4 ± 3.7

*Each value is expressed as a mean ± standard deviation (n = 3). **$p < 0.01$ vs. control, *$p < 0.05$ vs. control.

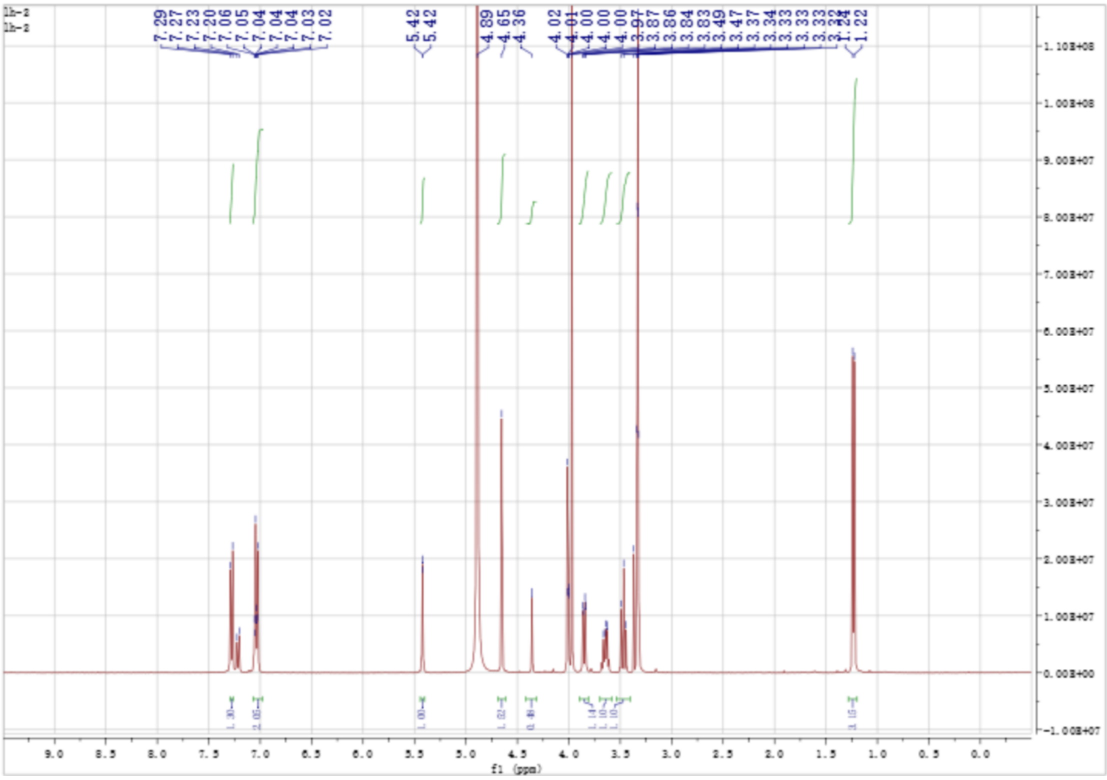

Fig. S1. The ^{1}H NMR spectrum (400 MHz, CD_3OD) of **1.**

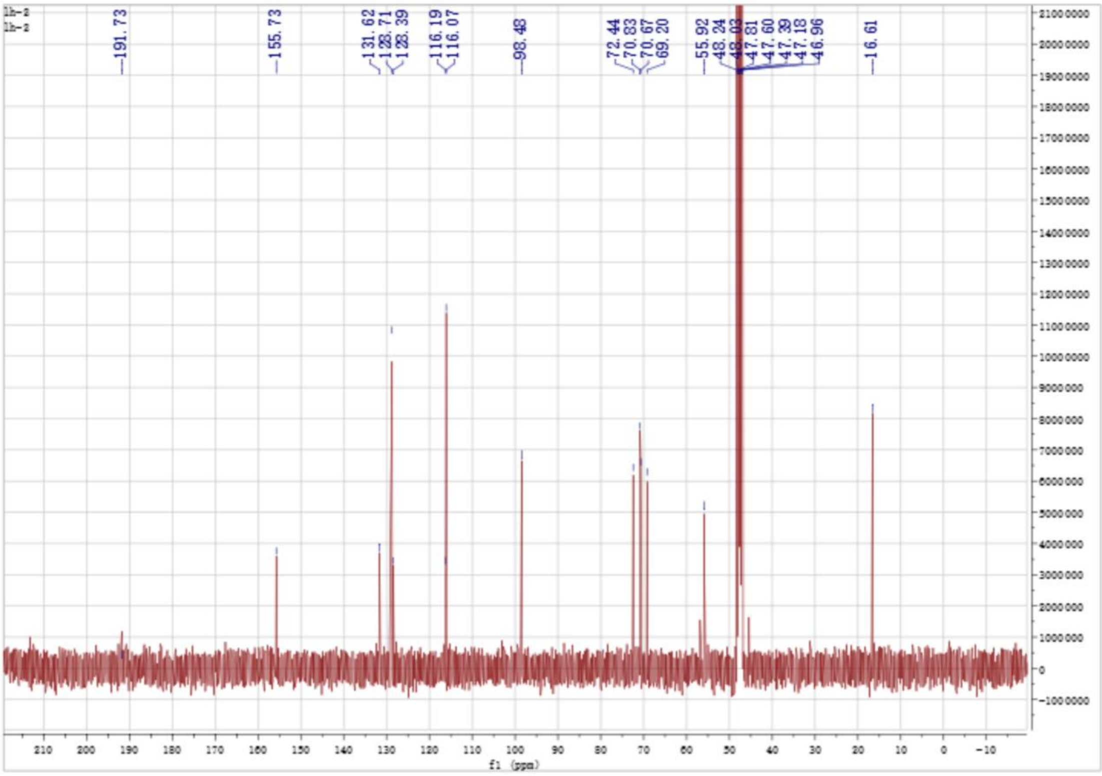

Fig. S2. The ^{13}C NMR (100 MHz, CD_3OD) of **1**

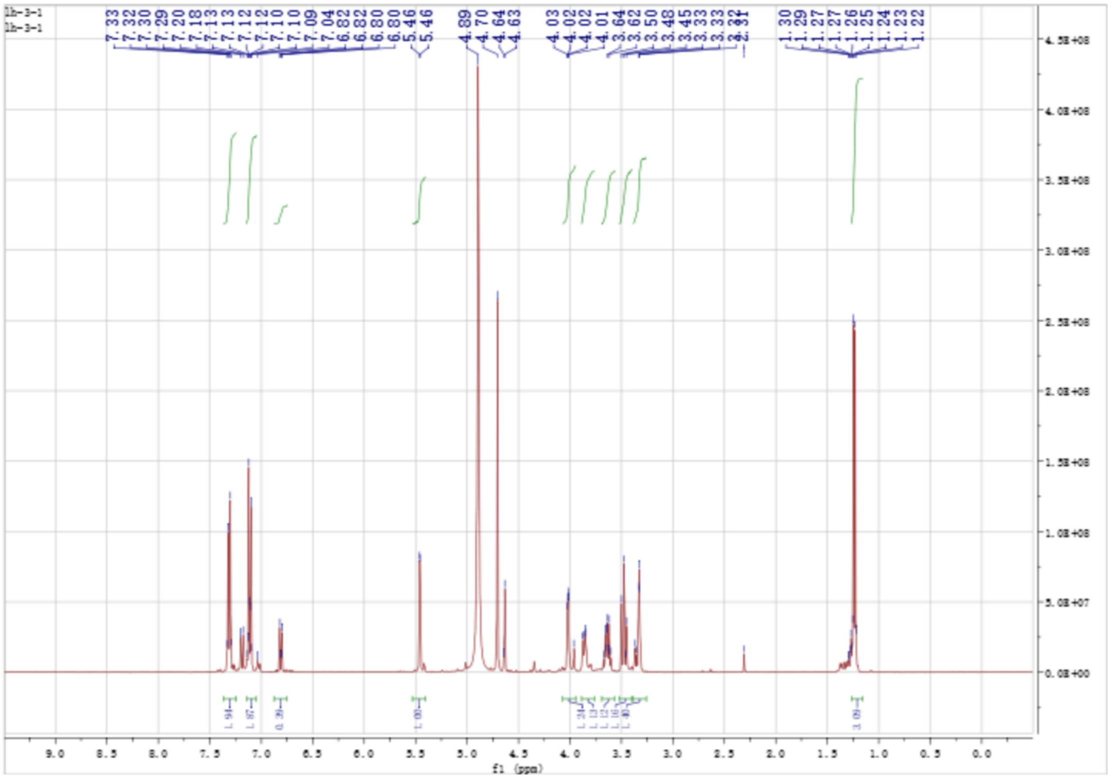

Fig. S3. The ^{1}H NMR spectrum (400 MHz, CD_3OD) of **2**.

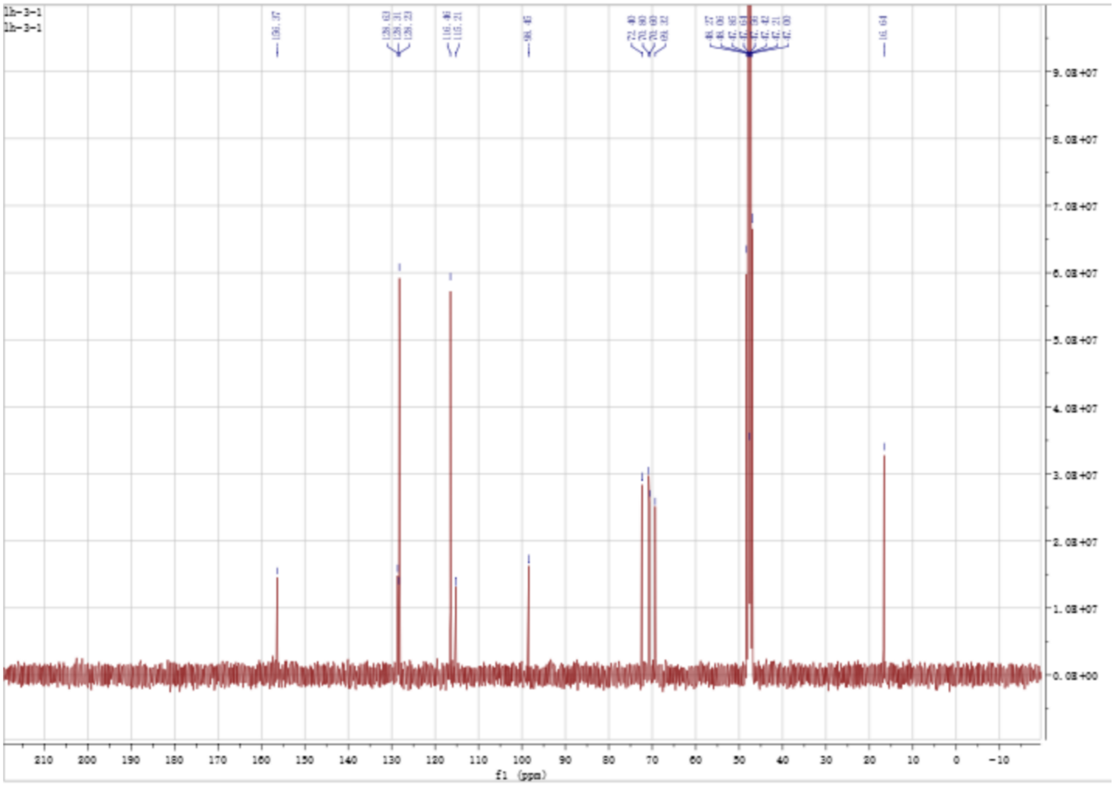

Fig. S4. The ^{13}C NMR (100 MHz, CD_3OD) of **2.**

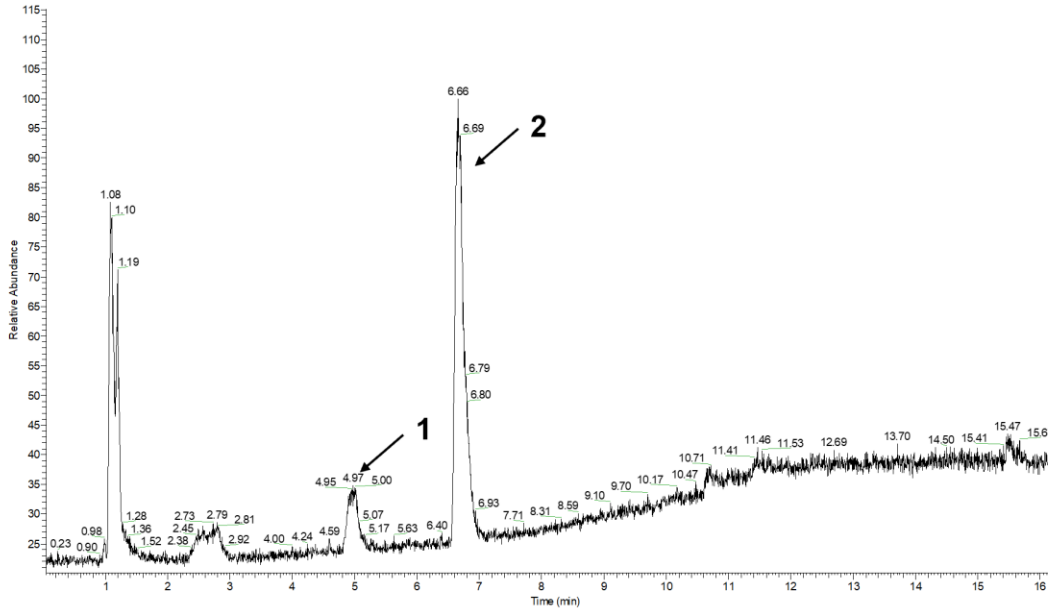

Fig. S5. Total ion chromatogram (TIC, negative ion mode) of the isolates (**1**and **2**) in *Moringa Oleifera* seeds extract.

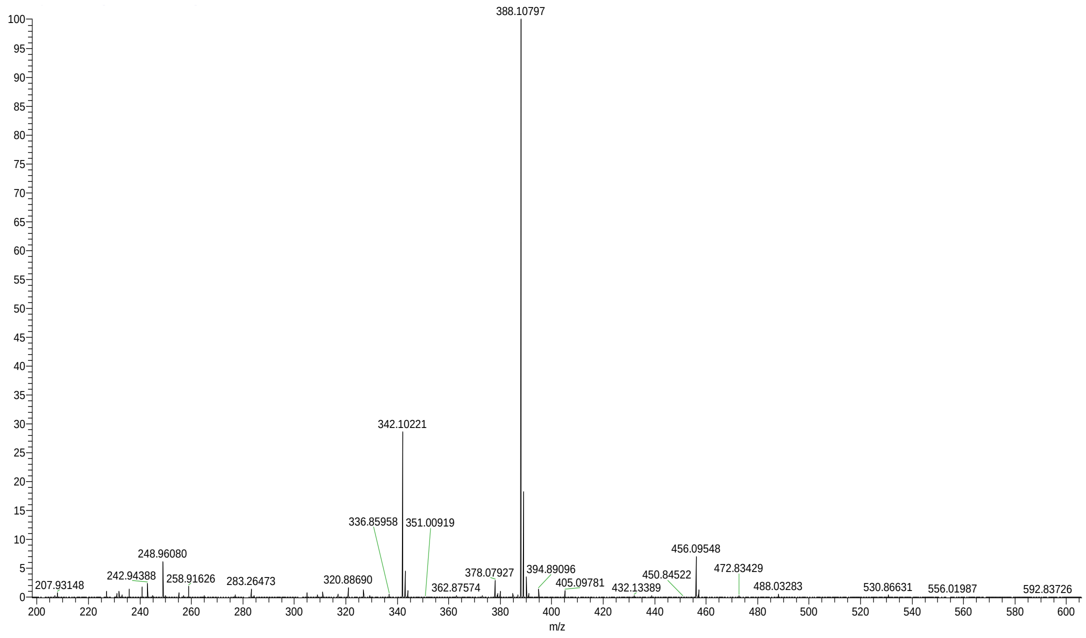

Fig. S6. The HR-ESI-MS (negative ion mode) spectrum of **1.**

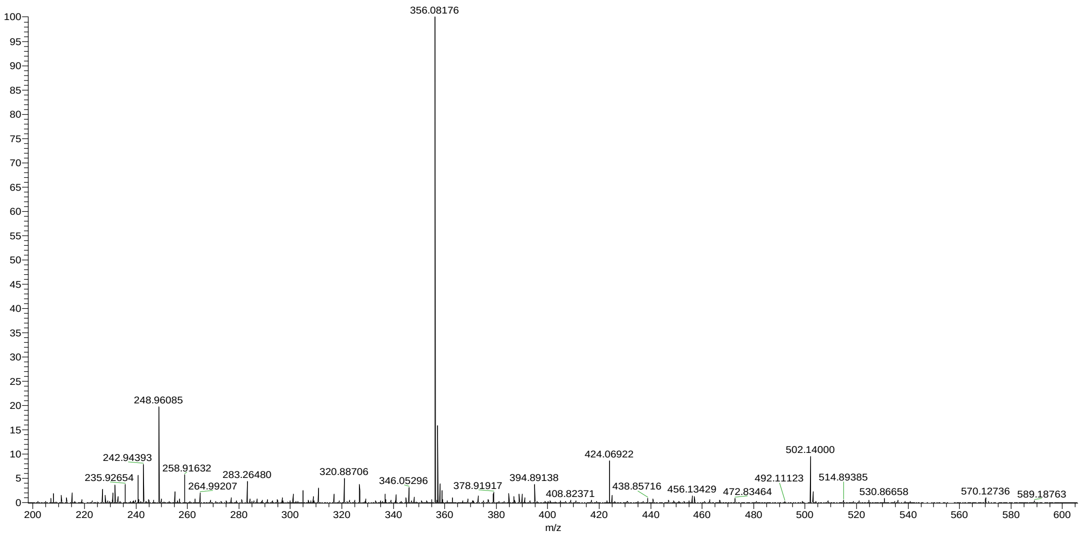

Fig. S7. The HR-ESI-MS (negative ion mode) spectrum of **2.**
